# The expanding role of fish models in understanding non-alcoholic fatty liver disease

**DOI:** 10.1242/dmm.011981

**Published:** 2013-05-29

**Authors:** Yoichi Asaoka, Shuji Terai, Isao Sakaida, Hiroshi Nishina

**Affiliations:** 1 Department of Developmental and Regenerative Biology, Medical Research Institute, Tokyo Medical and Dental University, 1-5-45 Yushima, Bunkyo-ku, Tokyo 113-8510, Japan; 2 Department of Gastroenterology and Hepatology, Yamaguchi University Graduate School of Medicine, Minami Kogushi 1-1-1, Ube, Yamaguchi 755-8505, Japan

## Abstract

Non-alcoholic fatty liver disease (NAFLD) is a condition in which excessive fat accumulates in the liver of an individual who has not consumed excessive alcohol. Non-alcoholic steatohepatitis (NASH), a severe form of NAFLD, can progress to hepatic cirrhosis and/or hepatocellular carcinoma (HCC). NAFLD is considered to be a hepatic manifestation of metabolic syndrome, and its incidence has risen worldwide in lockstep with the increased global prevalence of obesity. Over the last decade, rodent studies have yielded an impressive list of molecules associated with NAFLD and NASH pathogenesis. However, the identification of currently unknown metabolic factors using mammalian model organisms is inefficient and expensive compared with studies using fish models such as zebrafish (*Danio rerio*) and medaka (*Oryzias latipes*). Substantial advances in unraveling the molecular pathogenesis of NAFLD have recently been achieved through unbiased forward genetic screens using small fish models. Furthermore, these easily manipulated organisms have been used to great advantage to evaluate the therapeutic effectiveness of various chemical compounds for the treatment of NAFLD. In this Review, we summarize aspects of NAFLD (specifically focusing on NASH) pathogenesis that have been previously revealed by rodent models, and discuss how small fish are increasingly being used to uncover factors that contribute to normal hepatic lipid metabolism. We describe the various types of fish models in use for this purpose, including those generated by mutation, transgenesis, or dietary or chemical treatment, and contrast them with rodent models. The use of small fish in identifying novel potential therapeutic agents for the treatment of NAFLD and NASH is also addressed.

## Introduction

Over the past two decades, obesity has become a major public health challenge worldwide. It is clear that as-yet-unrecognized factors governing energy homeostasis must be uncovered in order to protect against the onslaught of metabolic diseases associated with excess adiposity (Dixon, 2010). In fact, the benefits gained from current therapies targeting obesity-related diseases (e.g. hypertension, coronary heart disease, hyperlipidemia and type 2 diabetes) are in danger of being outweighed by the negative effects of increased adiposity (Stewart et al., 2009). Thus, it is urgently necessary to develop systematic and comprehensive approaches to facilitate the identification of factors that play crucial roles in regulating energy homeostasis.

Among the obesity-related diseases, non-alcoholic fatty liver disease (NAFLD; see Box 1 for a full list of abbreviations) has garnered much attention from a broad range of researchers during the last decade. NAFLD is defined as the accumulation of fat in liver cells, known as fatty liver or hepatic steatosis, in the absence of excessive alcohol consumption. According to the practice guidelines of the American Association for the Study of Liver Diseases (AASLD), NAFLD is histologically subdivided into either non-progressive simple steatosis or a more severe condition called non-alcoholic steatohepatitis (NASH). NASH is a progressive chronic liver disease that is initially characterized by inflammation, fibrosis, and degenerative ‘ballooning’ of hepatocytes (Chalasani et al., 2012). NASH can eventually develop into life-threatening hepatic cirrhosis and/or hepatocellular carcinoma (HCC) after several decades. Indeed, the increased incidence of HCC in individuals with type 2 diabetes is most likely due to the high prevalence of NASH in this population (Ekstedt et al., 2006).

At present, our ability to treat NAFLD, particularly NASH, is constrained by our limited knowledge of the mechanisms underlying the progression of steatosis to more advanced liver inflammation and fibrosis. Researchers have therefore turned to studies of animal models of NAFLD, which are essential tools for gaining a full understanding of the pathophysiology of the human disease. Traditionally, rodent models have been used, and have not only proved helpful for revealing mechanisms underlying human NAFLD or NASH etiology but have also served as important platforms for testing the therapeutic potency of candidate agents (Nagarajan et al., 2012). However, although rodent models have greatly contributed to our understanding of human NAFLD, these results have come at a relatively high financial cost because these animals require considerable staff support and specialized infrastructure. These considerations have spurred the development of simpler and less expensive animal models to complement rodent-based research. Recent reports on energy homeostasis in worms, flies and small fish have shown that lower organisms can be used to accurately unravel metabolic processes underlying obesity in mammals (Schlegel and Stainier, 2007). Being vertebrates, small fish are structurally much more similar to humans than are worms and flies, and so have been exploited to successfully model various human diseases (Goldsmith and Jobin, 2012). In this Review, we discuss our current understanding of NAFLD (particularly NASH) pathogenesis as deduced from work with several fish models of this disease. We anticipate that insights emerging from these models will ultimately translate into the development of novel therapeutics for the treatment of severe human NAFLD.

Box 1. **Abbreviations**

**AASLD:** American Association for the Study of Liver Diseases

**Abcb7:** ATP-binding cassette sub-family B member 7

**ACC1:** acetyl CoA carboxylase 1

**ACO1:** acyl-CoA oxidase 1

**Ahcy:** S-adenosylhomocysteine hydrolase

**Apo:** apolipoprotein

**CB1R:** cannabinoid receptor 1

**C/EBP:** CCAAT/enhancer-binding protein

**Cdipt:** CDP-diacylglycerol-inositol 3-phosphatidyltransferase

**CHOP-10:** C/EBP homologous protein 10

**ChREBP:** carbohydrate response element-binding protein

**CPT1:** carnitine palmitoyltransferase 1

**DIO:** diet-induced obesity

**DNL:** *de novo* lipogenesis

**D-PAS:** diastase-periodic-acid-Schiff

**EPA:** eicosapentaenoic acid

**ER:** endoplasmic reticulum

**FAS:** fatty acid synthase

**FFA:** free fatty acid

**HBx:** hepatitis B virus X protein

**HCC:** hepatocellular carcinoma

**HCP:** hepatitis C virus core protein

**HFD:** high-fat diet

**IL:** interleukin

**MC4R:** melanocortin-4 receptor

**NAFLD:** non-alcoholic fatty liver disease

**NAS:** NASH activity score

**NASH:** non-alcoholic steatohepatitis

**NR1H3:** nuclear receptor subfamily 1 group H member 3

**OHdG:** 8-hydroxydeoxyguanosine

**PPAR:** peroxisome proliferator-activated receptor

**PUFA:** polyunsaturated fatty acid

**ROS:** reactive oxygen species

**SIRT1:** sirtuin-1

**SREBP:** sterol regulatory element-binding protein

**TAA:** thioacetamide

**TALEN:** transcription activator-like effector nuclease

**Tel:** telmisartan

**TILLING:** targeting induced local lesions in genomes

**TNF:** tumor necrosis factor

**TRAPPC11:** trafficking protein particle complex 11

**UPR:** unfolded protein response

**VLDL:** very low-density lipoprotein

**WT:** wild-type

**YY1:** yin yang 1

**ZFN:** zinc finger nuclease

## NASH pathogenesis: a ‘two-hit’ model

**Fig. 1.**
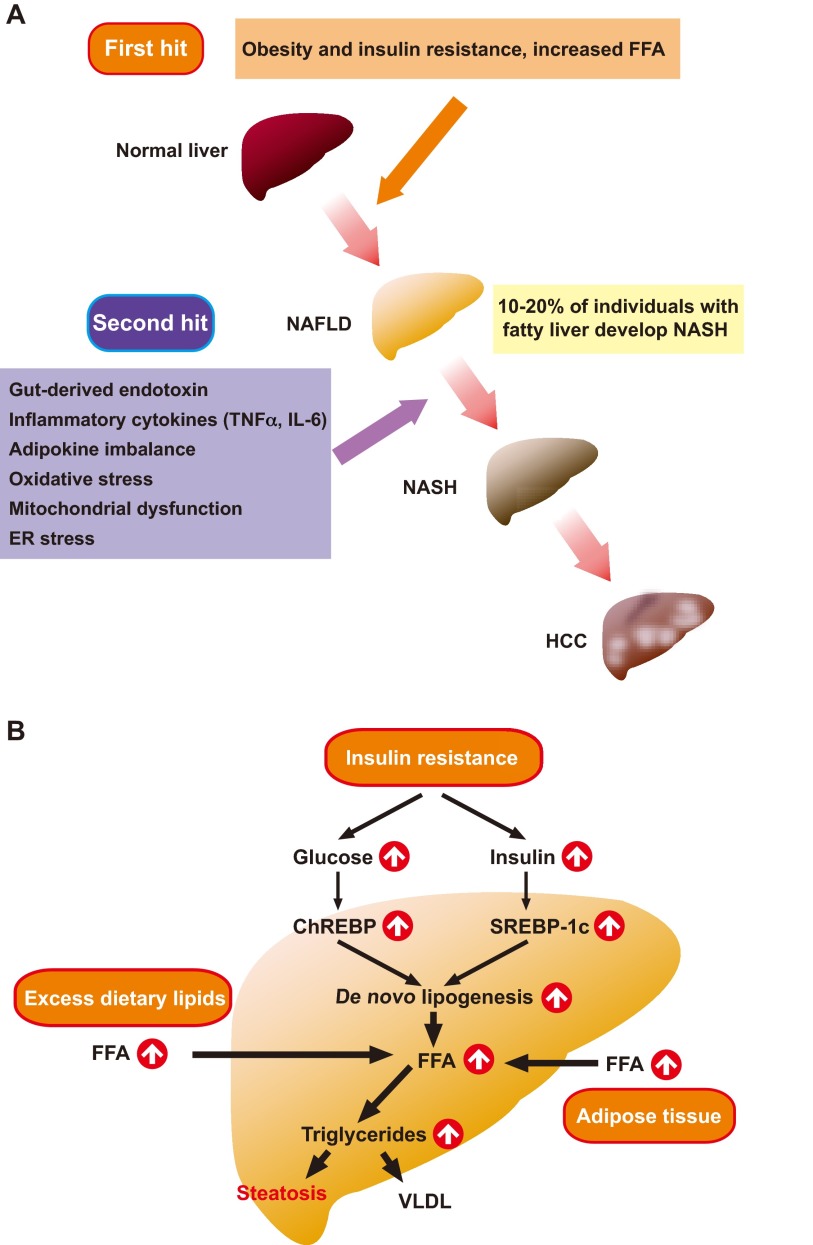
**Mechanisms of NASH: the two-hit model.** (A) In the two-hit hypothesis of liver disease development, steatosis (fatty liver causing NAFLD) represents the ‘first hit’ that sensitizes the liver to injury mediated by ‘second hits’, such as from endotoxin, inflammatory cytokines, adipokines, oxidative stress, mitochondrial dysfunction and/or ER stress. Receipt of both hits leads to the steatohepatitis and fibrosis of NASH, and eventually the development of hepatocellular carcinoma. (B) Obesity and insulin resistance lead to an increased release of FFA from adipose tissues and enhanced FFA flux to the liver. In the liver, hyperinsulinemia induces SREBP-1c expression, which increases *de novo* lipogenesis via activation of lipogenic gene transcription. At the same time, hyperglycemia activates ChREBP, which also activates the transcription of lipogenic genes, increasing *de novo* lipogenesis and FFA levels. These fatty acids can either be oxidized in the mitochondria to generate ATP, or esterified to produce triglycerides. These triglycerides are either incorporated into VLDL for export from hepatocytes, or are stored within hepatocytes, leading to steatosis.

The pathogenesis of NASH is thought to involve a two-step process in which the first ‘hit’ is excessive triglyceride accumulation in the liver that leads to NAFLD (Fig. 1A). The second ‘hit’, which results in NASH, is thought to involve additional pathogenic factors that can eventually induce liver damage, such as inflammatory cytokines, oxidative stress, mitochondrial dysfunction and/or endoplasmic reticulum (ER) stress (Day and James, 1998).

### The first ‘hit’

As summarized above, the initial step (NAFLD development) involves the hepatic accumulation of triglycerides. Triglycerides are produced by the esterification of free fatty acids (FFAs) and glycerol within hepatocytes (Fig. 1B). FFAs are derived from three distinct sources: (1) circulating fatty acids released from adipose tissue, (2) dietary sources, and (3) *de novo* lipogenesis (DNL) (Postic and Girard, 2008). FFAs can be metabolized by one of two pathways: β-oxidation to generate ATP, or esterification to produce triglycerides. These triglycerides are either stored in lipid droplets within hepatocytes, or are packaged and released as very low-density lipoprotein (VLDL) particles into the blood. In the fasting state, the contribution of DNL to the hepatic triglyceride pool is normally quite low. However, DNL is highly elevated in hepatocytes of individuals who are insulin-resistant and have NAFLD (Schwarz et al., 2003). This insulin resistance is manifested as hyperinsulinemia and hyperglycemia. In the livers of these individuals, hyperinsulinemia leads to the upregulation of SREBP-1c, the master transcriptional regulator of all lipogenic genes. Simultaneously, hyperglycemia activates carbohydrate-responsive element binding protein (ChREBP), which transcriptionally activates genes that are involved in DNL and thus promotes an imbalance in lipid input relative to output; this imbalance then results in hepatic steatosis (Fig. 1B).

### The second ‘hit’

#### The role of inflammatory cytokines and adipokines

The progression of NAFLD to NASH is largely driven by liver Kupffer cells, which secrete a variety of inflammatory cytokines. TNFα production is one of the earliest events in liver injury and triggers the secretion of other cytokines that recruit inflammatory cells, kill hepatocytes and initiate fibrogenesis. In rodent studies, TNFα was shown to be dysfunctionally released when animals exhibited hepatic steatosis, and this TNFα contributed to NASH severity (Diehl, 2002). Hepatic levels of TNFα are consistently elevated in humans with NASH and again correlate with histological severity. Furthermore, emerging evidence suggests that inflammation in any tissue can promote carcinogenesis, and that the chronic inflammatory state associated with hepatic steatosis might play a crucial role in HCC progression (Day, 2006).

In addition to cytokines released by the liver, cytokines synthesized by adipose tissues can contribute to NASH. Adipokines are a subset of adipose-tissue-derived cytokines whose functional roles in NAFLD have recently been recognized. For example, leptin is an adipokine secreted mainly by mature adipocytes. The functions of leptin include the regulation of energy intake and consumption, modulation of the immune system, and induction of inflammatory and fibrogenic signals. Elevated leptin levels are frequently observed in individuals with NAFLD, which is now deemed to be a leptin-resistant condition (Huang et al., 2008). Some researchers therefore believe that leptin plays a key role in NAFLD development.

Adiponectin is another well-studied adipokine that exerts anti-inflammatory effects and increases insulin sensitivity. The secretion and circulating levels of adiponectin are inversely correlated with body fat content and are decreased in individuals with NAFLD (Bugianesi et al., 2005). In murine models of NAFLD, administration of adiponectin suppresses liver enlargement and improves the biochemical and histological signs of NAFLD (Tomita et al., 2008; Xu et al., 2003). Furthermore, adiponectin attenuates the effects of TNFα, which reciprocally suppresses adiponectin production (Whitehead et al., 2006). The importance of adiponectin in NAFLD is supported by studies indicating that serum adiponectin levels can help to predict the severity of liver injury in individuals with NAFLD (Lemoine et al., 2009).

#### Oxidative stress and mitochondrial dysfunction

Recent studies have revealed the important roles of oxidative stress and subsequent mitochondrial dysfunction in NAFLD and NASH, with the stage and grade of NAFLD correlating with the level of oxidative stress (Chalasani et al., 2004). Normally, fatty-acid β-oxidation within the liver occurs in the mitochondria. However, in the context of NAFLD, this process can become impaired as a result of the increased FFA load, leading to the generation of reactive oxygen species (ROS) (Sanyal et al., 2001). ROS can cause oxidative stress, which in turn upregulates inflammatory pathways and causes mitochondrial damage. Structural mitochondrial abnormalities (enlarged mitochondria, loss of mitochondrial cristae and presence of paracrystalline inclusion bodies) and impaired mitochondrial electron transport chain enzyme activity have been observed in humans with NASH (Pessayre and Fromenty, 2005).

#### ER stress

ER stress is another pathogenic mechanism implicated in NASH development (Day, 2006). The ER is one of the largest cellular organelles and serves many specialized functions, including the translocation of secretory proteins across the ER membrane, insertion of membrane proteins, protein folding and modification in the ER lumen, phospholipid biosynthesis, and detoxification. The disruption of ER homeostasis constitutes ER stress, which can be induced by a variety of biological insults such as hyperinsulinemia and hyperlipidemia. ER stress can subsequently upregulate various signaling pathways, leading to insulin resistance, inflammation, mitochondrial dysfunction and apoptosis (Day, 2006). The involvement of ER stress in NAFLD is less well studied than in obesity, diabetes and cardiovascular disease, despite evidence that it plays a part in NAFLD pathogenesis (Puri et al., 2008).

## Fish models of NAFLD and NASH

### Advantages of fish models

As noted above, rodents are the traditional organisms used to uncover mechanisms underlying diseases of the liver, as well as to establish diagnostic criteria and medical therapies for individuals with liver disease. For example, melanocortin-4-receptor-deficient (MC4R-KO) mice can be induced to develop a liver condition that is similar to human NASH (Itoh et al., 2011). MC4R is a seven-transmembrane G-protein-coupled receptor that is expressed by cells in the hypothalamus and is involved in the control of feeding behavior and body weight (Balthasar et al., 2005). After 20 weeks of feeding on a high-fat diet (HFD), MC4RKO mice exhibit obesity, insulin resistance and dyslipidemia. These mutants then develop multiple HCCs if maintained on the HFD for another 7 months. Thus, this strain has been used to investigate the sequence of events leading to hepatic steatosis, liver fibrosis and HCC (Itoh et al., 2011). However, like all rodent models, the use of the MC4R-KO mouse is hampered by certain limitations that do not apply to fish models of this disease. Rodent models are not necessarily suited to large-scale drug screening because these animals have a relatively large body size, produce small litters and incur high husbandry costs. Furthermore, to examine their livers for abnormalities, rodents must be sacrificed and their internal organs surgically isolated. This requirement does not allow for parallel, continuous, real-time monitoring of liver condition in multiple animals.

Small fish such as zebrafish (*Danio rerio*) and medaka (*Oryzias latipes*) have a short generation time, are highly fertile, and cost little in terms of housing space and daily maintenance owing to their tiny size. They are also much easier to screen for abnormal phenotypes because their larvae are optically clear and their internal organs can be directly observed without the need for surgery. Thus, real-time, simultaneous monitoring of livers in multiple animals is easily achieved. Zebrafish and medaka have therefore been increasingly used as alternatives to rodents for vertebrate developmental and toxicological studies, as well as for drug screening and evaluation programs. In addition, lipids can be visualized directly in live zebrafish using fluorescent fatty-acid analogs (Carten et al., 2011), making these small fish powerful models for studying lipid-related diseases such as NAFLD. We and other groups have successfully elucidated various aspects of NAFLD and NASH induction by using zebrafish and medaka that have naturally occurring or engineered mutations of certain genes, or express engineered transgenes. Alternatively, these fish have been subjected to HFD feeding or treatment with a carcinogenic chemical compound to induce NAFLD or NASH. In the following sections, we review the results of research that has made use of these approaches.

## Mutant fish models of NAFLD and NASH

At present, several fish mutants with hepatic steatosis have been characterized. The affected genes and phenotypes of these animals are summarized in Table 1.

**Table 1.**
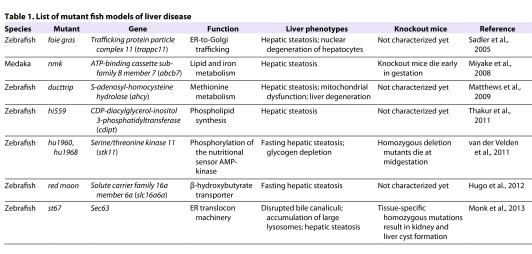
List of mutant fish models of liver disease

### *foie gras *(*fgr*) zebrafish

The *foie gras* (*fgr*) zebrafish mutant was the first fish to be reported as a model of hepatic steatosis (Sadler et al., 2005). This mutant was identified in a screen for hepatomegaly at day 5 of embryogenesis. The livers of *fgr* mutants exhibit enlarged hepatocytes, hepatocyte nuclear degeneration and steatosis. Thus, *fgr* fish display most of the hallmarks of mammalian NASH, with the exception of inflammation. These *fgr* defects can be phenocopied by a translation-blocking morpholino, suggesting that *fgr* is a null mutation. The Fgr protein is the zebrafish ortholog of human TRAPPC11 (trafficking protein particle complex 11), which is involved in ER-to-Golgi trafficking (Scrivens et al., 2011). Interestingly, Cinaroglu et al. found that the *fgr* mutation causes hepatic ER stress that is linked to hepatic steatosis (Cinaroglu et al., 2011). Furthermore, this ER stress causes hepatocyte apoptosis that is controlled in part through the *atf6* gene, which is involved in the unfolded protein response (UPR). Morpholino blockade of *atf6* can prevent the liver injury that occurs in *fgr* mutants experiencing chronic ER stress. However, *atf6* blockade potentiates the steatosis that develops in wild-type (WT) zebrafish when acute ER stress is induced by treatment with the toxin tunicamycin (Cinaroglu et al., 2011). These results suggest that Atf6 might have different effects in acute and chronic phases of liver injury.

### *namako *(*nmk*) medaka

A screen designed to isolate medaka mutants with abnormal livers identified the *namako* (*nmk*) mutant (Miyake et al., 2008). The liver in *nmk* medaka develops normally until hatching but, by 2 days post-hatching, this organ exhibits altered morphology and loss of transparency. The mutant hepatocytes are arranged in a random manner and vacuoles are plentiful, evoking the pathology of rodent hepatic steatosis. Furthermore, the mitochondria in *nmk* livers tend to be enlarged and swollen, a characteristic of fatty liver. Positional cloning of the gene responsible for the *nmk* phenotype revealed that a mutation in the ATP-binding cassette sub-family B member 7 gene (*abcb7*) causes an Asp substitution at the Val residue in position 219, which is an amino acid conserved among most vertebrate species. The expression of genes involved in iron and lipid metabolism is also affected in *nmk* liver. Among these genes, *apoliporotein* (*apo*) *B-100* and *apoE* are thought to play important roles in the pathogenesis of fatty liver. These proteins are both components of VLDL and positively control its production rate. Thus, ApoB-100 and ApoE are rate-determining factors in hepatocyte-lipid export. In line with this, *apoE* deficiency in mice leads to hepatic steatosis (Mensenkamp et al., 2000).

### *ducttrip* (*dtp*) zebrafish

The *ducttrip* (*dtp*) zebrafish mutant was originally isolated in a chemical mutagenesis screen for defects in exocrine pancreas development (Yee et al., 2005). Subsequent experiments revealed that the *dpt* mutant displays hepatic steatosis, mitochondrial dysfunction and liver degeneration (Matthews et al., 2009). Positional cloning identified a causative mutation in the *ahcy* gene, which encodes S-adenosylhomocysteine hydrolase. This enzyme is crucial for the hydrolysis of S-adenosylhomocysteine to homocysteine and adenosine, a pathway whose disruption has been linked to mitochondrial dysfunction and hepatic steatosis. The *dtp* mutant shows elevated expression of *TNFα* and *PPARγ* genes, which is thought to occur via an epigenetic mechanism involving changes in histone methylation coding (Matthews et al., 2009). Such changes can trigger an inflammatory reaction marked by enhanced TNFα production.

### *hi559* zebrafish

The signaling pathways that maintain hepatic homeostasis are highly conserved, as demonstrated by studies of the *hi559* zebrafish mutant. The *hi559* mutant has an inactivating insertion in the *CDP-diacylglycerol-inositol 3-phosphatidyltransferase* (*cdipt*) gene (Thakur et al., 2011). This mutation eliminates phosphatidyl inositol synthesis, and the lack of this crucial phospholipid in the liver seems to induce persistent hepatocyte ER stress marked by activation of the UPR. Concurrent with the presence of ER stress, the *hi559* liver displays NAFLD pathologies, including macrovesicular steatosis, hepatocyte ballooning and necroapoptosis. Subsequent analysis of the ultrastructural pathology of *hi559* hepatocytes has demonstrated that the morphology of their mitochondria is abnormal, although it is unknown whether this structural change affects fatty-acid β-oxidation (Thakur et al., 2011). The *hi559* mutant was the first *in vivo* model linking phosphatidyl inositol synthesis, ER stress and NAFLD. As such, this finding confirms that additional work investigating the requirement for phosphatidyl inositol in molecular pathways of normal hepatic lipid metabolism is merited.

## Transgenic fish models

Several research groups have attempted to create NAFLD and NASH models in small fish using transgenic approaches (Table 2).

**Table 2.**
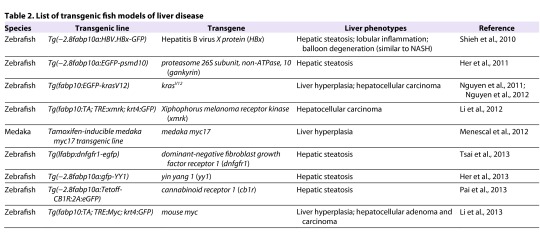
List of transgenic fish models of liver disease

### HBx zebrafish

Shieh et al. demonstrated that engineered expression of the hepatitis B virus X protein (HBx) in zebrafish induces hepatic steatosis by upregulating C/EBP-α, SREBP1 and ChREBP, three important adipogenic genes that drive *de novo* FFA synthesis (Shieh et al., 2010). Histological examination of the livers of multiple independent HBx transgenic lines showed that they all exhibited hepatic steatosis, lobular inflammation and hepatocyte ballooning. This study was the first report demonstrating that forced expression of HBx *in vivo* can cause a wide range of liver disease phenotypes in lower vertebrates.

### Gankyrin, YY1 and CB1R zebrafish

Three transgenic zebrafish models of hepatic steatosis have been generated by the group of Her (National Taiwan Ocean University). In the first of these models, hepatic lipid accumulation was studied in *gankyrin* transgenic zebrafish (Her et al., 2011). Gankyrin is a small ankyrin-repeat protein previously shown to be involved in normal cellular proliferation as well as in HCC tumorigenesis (Higashitsuji et al., 2000). Over 90% of viable adult *gankyrin* transgenic zebrafish exhibit an increase in hepatic lipid content that leads to liver steatosis. Molecular analysis revealed that *gankyrin* overexpression induces hepatic steatosis and modulates the expression profiles of four hepatic microRNAs involved in lipid metabolism. This study was the first to demonstrate that *gankyrin* overexpression is pathological and induces the development of liver steatosis via dysregulation of hepatic microRNAs. In the two other transgenic zebrafish models described by Her’s group, hepatic steatosis is induced by liver-specific overexpression of yin yang 1 (YY1) or by cannabinoid receptor 1 (CB1R) (Her et al., 2013; Pai et al., 2013). YY1 induces the expression of important lipogenic genes, such as *C/EBP-α*, *PPARγ* and *SREBP-1c*, by inhibiting C/EBP homologous protein 10 (CHOP-10) expression, whereas CB1R stimulates the expression of *SREBP-1c*. In turn, the upregulation of those lipogenic factors drives DNL, which is expected to lead to hepatic accumulation of FFA and triglycerides.

### *Lfabp:dnfgfr1-egfp* zebrafish

Another transgenic zebrafish that is used to study hepatic steatosis was generated by expressing a dominant-negative Fgf receptor mutation specifically in hepatocytes (*lfabp:dnfgfr1-egfp*) (Tsai et al., 2013). Hepatocytes in young *lfabp:dnfgfr1-egfp* zebrafish display hepatocyte ballooning, whereas adult fish display hepatic steatosis and cholestasis. These findings confirm that liver homeostasis is dysregulated when Fgf signaling is repressed. This analysis closely paralleled the mouse studies of Steiling et al., which demonstrated that hepatocellular expression of dominant-negative FGFR2b accelerates the development of fatty liver in aged transgenic animals (Steiling et al., 2003). This similarity of results achieved in mouse and fish models reinforces the validity of exploiting the latter to study NAFLD. In particular, the genetic tractability of fish and the ability to examine the entire pathological process *in vivo* are particular strengths of small fish models of hepatic steatosis.

## Dietary fish models

### HFD-medaka

Our group has developed a NASH model in medaka that is based on feeding the fish a HFD (Matsumoto et al., 2010) (Table 3). Medaka that consume a HFD (HFD-medaka) exhibit hyperlipidemia, hyperglycemia and the hepatocyte ballooning that is a key diagnostic feature of human NASH (Brunt et al., 1999; Matteoni et al., 1999; Neuschwander-Tetri and Caldwell, 2003). The frequency of ballooning in WT medaka is 10–20% (Boorman et al., 1997; Brown-Peterson et al., 1999; Bunton, 1990), whereas over 60% of HFD-medaka exhibited this phenotype. In contrast, it was reported that only 46% of C57BL/6 mice fed a HFD develop steatohepatitis (Deng et al., 2005). Therefore, the medaka model of NASH is slightly superior to the rodent model (64% vs 46% efficiency) in terms of inducing the ballooning degeneration of hepatocytes typical of this disease.

**Table 3.**
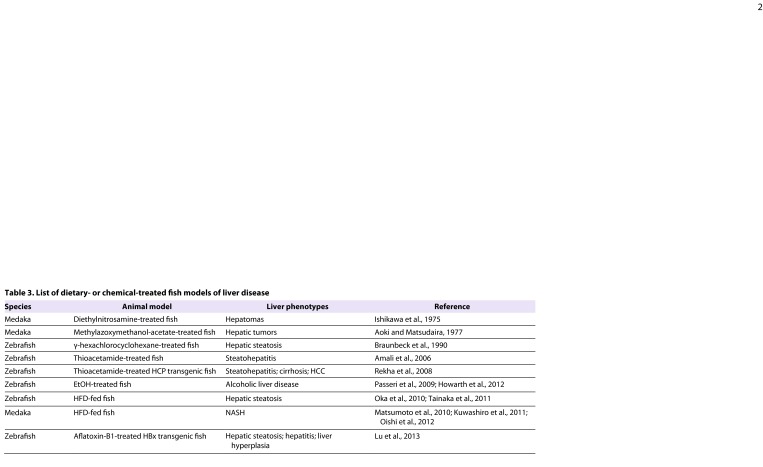
List of dietary- or chemical-treated fish models of liver disease

Hepatocytes of HFD-medaka show increased expression of lipogenic genes (*SREBP-1c*, *FAS* and *ACC1*) and decreased expression of lipolytic genes (*PPARα* and *CPT1*). These data suggest that HFD-medaka suffer from increased fatty-acid synthesis accompanied by decreased fatty-acid β-oxidation and inflammation, accounting for their fatty livers. Previously, it was reported that treatment with n-3 polyunsaturated fatty acid (PUFA), a mixture of eicosapentaenoic acid (EPA) and docosahexaenoic acid, improves features of hepatic steatosis and necroinflammation in humans (Capanni et al., 2006). The EPA-evoked anti-inflammatory effects are thought to result from mechanisms involving decreased FFA-derived lipotoxicity in liver cells (Sekiya et al., 2003), and administration of EPA has been investigated as a therapy for human NASH (Tanaka et al., 2008). In light of these findings, we explored whether EPA treatment could suppress disease progression in our medaka NASH model. Treatment of HFD-medaka with EPA alleviates the disease phenotypes, as judged by the recovery of normal liver fatty-acid composition and normal expression levels of lipogenic and lipolytic genes. In addition, medaka fed an n-3 PUFA-deficient diet develop NASH features. Thus, NASH can be induced in medaka by a HFD, and the proportion of n-3 PUFAs in the liver affects NASH pathogenesis in these fish. Subsequent work has confirmed that this medaka NASH model is helpful for advancing our understanding of human NASH pathology and can assist in the clinical development of novel therapeutics, as described below (Kuwashiro et al., 2011; Oishi et al., 2012).

### DIO-zebrafish

**Fig. 2.**
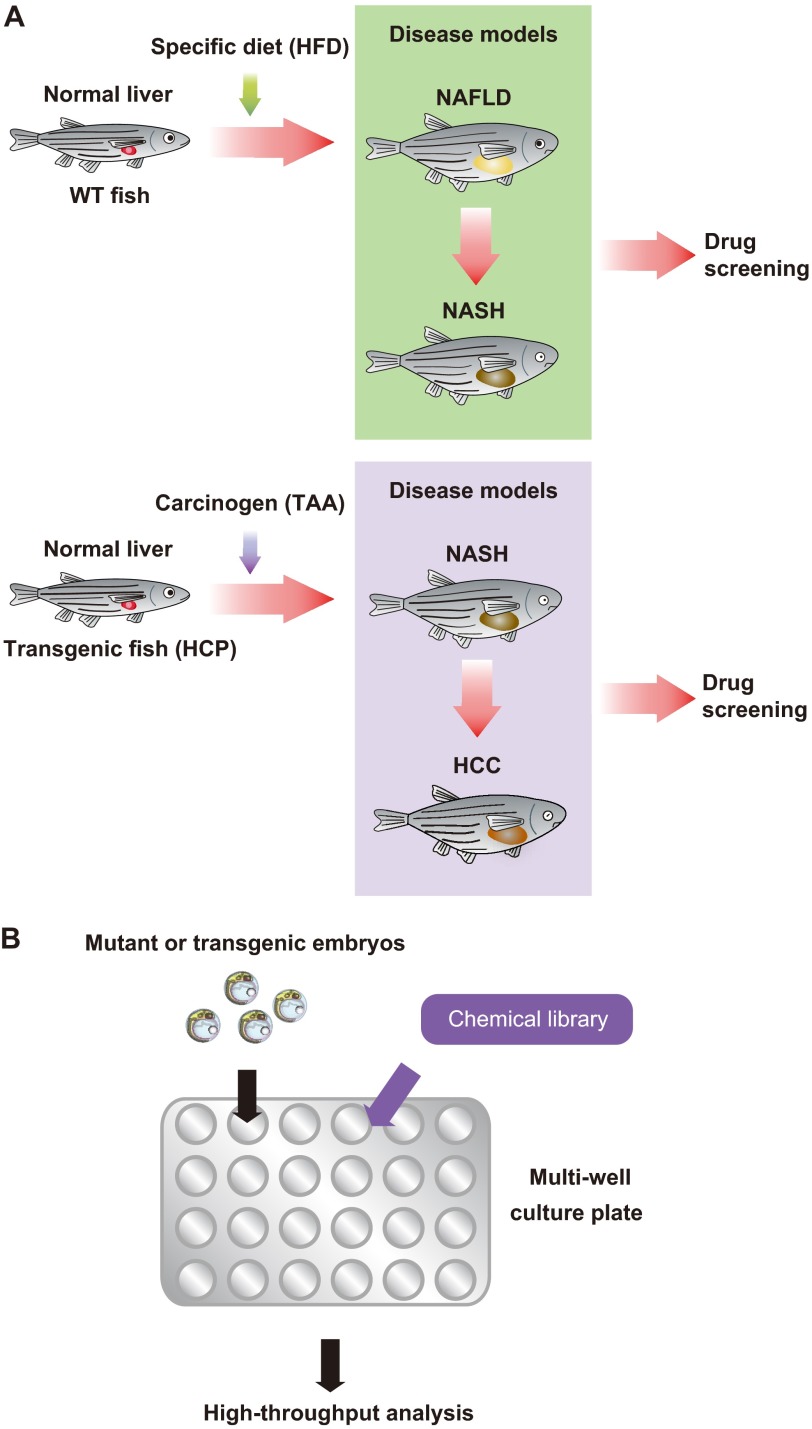
**Drug screening strategy using fish disease models.** (A) An overview of the NAFLD, NASH and HCC drug screening strategy in fish models. WT or transgenic (HCP) fish are raised on a specific diet (HFD) or treated with a specific carcinogen (TAA), giving rise to NAFLD and NASH, or NASH and HCC, respectively. The disease models are exposed to candidate drugs to determine whether the development of NAFLD, NASH or HCC can be mitigated. (B) Small-molecule inhibitor screen. Chemical libraries can be aliquoted to multi-well plates that contain fish growth medium. Large numbers of mutant or transgenic fish can be mated to generate thousands of embryos, which are placed in the multi-well plates containing medium and test reagents. These multi-well plates easily allow the detailed observation of embryo morphology under a dissecting microscope. To investigate chemical-induced changes in specific markers, several techniques have been established to perform high-throughput analysis, such as whole-mount immunohistochemistry or whole-mount *in situ* hybridization on large numbers of embryos.

Oka et al. have used an alternative dietary approach to establish a zebrafish model of diet-induced obesity (DIO) with NAFLD features (Oka et al., 2010). Zebrafish overfed with *Artemia* (DIO-zebrafish) exhibit increased body mass index, hypertriglyceridemia and hepatic steatosis. Comparative transcriptome analysis of visceral adipose tissue revealed that the pathophysiological pathways associated with mammalian obesity are similarly altered in DIO-zebrafish. In both DIO-zebrafish and obese mammals, dysregulation of genes involved in the blood coagulation pathway, such as *apoH*, *interleukin-6* (*IL-6*) and *IL-1β*, and genes involved in lipid metabolism, such as *SREBP1*, *PPARα/γ*, *NR1H3* and *leptin*, has been observed. These findings suggest that DIO-zebrafish, like HFD-medaka, could be useful for identifying putative pharmacological targets and testing novel chemical compounds for the treatment of human hepatic steatosis (Fig. 2).

## Chemically treated fish models

In Japan, the history of medaka as a research organism began in the 1970s with the investigation of their use as a liver tumor model. Indeed, medaka proved to be an organism that is sensitive to many carcinogens and is thus highly useful for tumorigenesis research (Aoki and Matsudaira, 1977; Ishikawa et al., 1975). Starting in the 1990s, models of hepatic steatosis were established in zebrafish by treating them with a potent hepatotoxic agent such as γ-hexachlorocyclohexane or thioacetamide (TAA) (Table 3) (Amali et al., 2006; Braunbeck et al., 1990). Subsequently, Rekha et al. reported that zebrafish that overexpress hepatitis C virus core protein (HCP) and are treated with TAA develop HCCs faster than standard mouse models (Rekha et al., 2008). The speed of this particular zebrafish HCC model could make it a powerful platform for screening for drugs that are effective against HCCs.

## Screening for drugs relevant to NAFLD

In addition to advancing basic research into NAFLD mechanisms, small fish have proved useful in novel assay systems designed to screen for new candidate drugs for these disorders (Fig. 2A). Several pharmaceutical companies have developed drug discovery methodologies based on high-throughput *in vivo* drug screening using small fish models of human diseases. The HFD-medaka NASH model (Matsumoto et al., 2010) has been particularly helpful for the investigation of candidate drug effectiveness (Table 4), owing to its similarity to human NAFLD (particularly NASH).

**Table 4.**
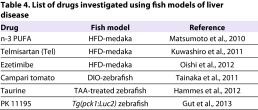
List of drugs investigated using fish models of liver disease

### Insights into drug efficacy from the HFD-medaka model

In 2011, our group published a study in which we used our HFD-medaka NASH model to investigate the efficacy of the anti-hypertensive drug telmisartan (Tel) (Kuwashiro et al., 2011). This drug blocks angiotensin-II type 1 receptors, excites PPARγ, and reportedly improves glycolipid metabolism in humans. Using the NASH activity score (NAS), a classification system for human NASH pathology in clinical settings (Kleiner et al., 2005), we assessed liver histology in our HFD-medaka NASH model and confirmed that the NAS increases over time in the absence of Tel. Treatment of HFD-medaka with Tel results in an increase in the expression of liver *PPARγ*, *CPT1* and *ACO1*, a decrease in the number of 8-hydroxydeoxyguanosine (OHdG**)**-positive hepatocytes in livers, and a reduction in the infiltration of macrophages that are positive for diastase-periodic-acid-Schiff (D-PAS). Thus, Tel administration might induce the β-oxidation of FFAs in the liver of HFD-medaka and thereby contribute to the reduction of triglycerides in this organ. Interestingly, the fatty-acid content of the liver in these fish is not affected. These results demonstrate that Tel administration can ameliorate NASH pathology in HFD-medaka and improve their NAS values.

We have also examined the effects of ezetimibe in our HFD-medaka NASH model (Oishi et al., 2012). Ezetimibe is a small-molecule inhibitor of the intestinal cholesterol transporter encoded by the Niemann-Pick C1-like 1 (*NPC1L1*) gene and is used to treat individuals with high blood cholesterol levels. We found that ezetimibe treatment of HFD-medaka reduces total cholesterol and triacylglycerol in the blood, as expected. However, ezetimibe also induces a significant decrease in fatty acids in the liver. The expression levels of genes related to hepatic fatty-acid metabolism are also reduced by ezetimibe administration. Histological examination of the livers of ezetimibe-treated HFD-medaka revealed reductions in the number of inflammatory cells and the NAS value. This decrease in liver fatty acids indicates that the NASH symptoms in HFD-medaka can be mitigated by ezetimibe, a finding with potential implications for treatment of the human disease.

### Insights into drug efficacy from zebrafish models

Recently, several epidemiological studies have demonstrated that high dietary intake of vegetable products is beneficial against obesity and its related diseases. For example, tomato and its components were shown to lower plasma cholesterol and triacylglyceride and prevent obesity-related diseases, including hypertension in humans (Engelhard et al., 2006) and NASH-promoted hepatocarcinogenesis in rats (Wang et al., 2010). In 2011, Tainaka et al. used DIO-zebrafish to evaluate the influence of certain food components on the development of human hepatic steatosis. Zebrafish are polyphagous animals and readily eat human foods, making them highly suitable for feeding experiments designed to perform *in vivo* screening of orally administered test compounds. In this particular study, DIO-zebrafish were subjected to transcriptomic analysis to test the effects on gene expression of certain vegetables (Tainaka et al., 2011). Among all vegetables tested, Campari tomato suppressed diet-induced obesity in zebrafish, as judged by reductions in dyslipidemia and hepatic steatosis, and the downregulation of lipogenic genes such as *SREBP1*. This is the first study to use zebrafish for food component evaluation, and its results indicate that DIO-zebrafish are a powerful tool for identifying foods that can serve as natural medicines for the prevention or treatment of human hepatic steatosis.

Zebrafish have also been used to test taurine (2-aminoethanesulfonic acid) as a hepatoprotective agent (Hammes et al., 2012). Taurine has been hypothesized to protect against TAA-mediated hepatic steatosis because of its hypolipidemic and antioxidant effects (Balkan et al., 2001). Compared with zebrafish treated with TAA alone, TAA-treated zebrafish that also receive taurine show reductions in liver lipid accumulation and oxidative stress parameters. These findings are in agreement with the rodent model results of Chen et al., which showed that oxidative stress is decreased and histological parameters of hepatic steatosis are improved in NASH-affected rats that are treated with taurine (Chen et al., 2006). Additional examination of taurine+TAA-treated zebrafish has revealed that taurine decreases hepatic steatosis by increasing *sirtuin-1* (*sirt1*) mRNA expression. SIRT1 is a nicotinamide adenine dinucleotide (NAD^+^)-dependent deacetylase that has recently been shown to protect against NAFLD pathogenesis (Colak et al., 2011). The study of Hammes et al. is the first to evaluate the effect of taurine on SIRT1 expression in hepatic steatosis (Hammes et al., 2012). On the basis of these results, taurine might be a promising therapy for human NASH.

Gut et al. have recently described an innovative drug discovery strategy in which transgenic reporter zebrafish were used to identify small molecules that can modulate the expression of the fasting-inducible gluconeogenic gene *pck1* (Gut et al., 2013). Transgenic zebrafish expressing a bioluminescence reporter gene under the control of the *pck1* promoter were generated and treated with drugs known to affect gluconeogenesis in humans, as well as with several metabolically uncharacterized compounds. It was found that the translocator protein ligands PK 11195 and Ro5-4864 decrease glucose levels in the reporter fish despite a strong inductive effect on the *pck1* promoter. Notably, when translated to a mammalian system, PK 11195 treatment prevents the hepatic steatosis and glucose intolerance that develop in obese mice fed a HFD (Gut et al., 2013). These results validate the zebrafish data in the mammalian context and help to build a framework that is suitable for developing a new class of drugs for metabolic diseases.

## Concluding remarks

Zebrafish and medaka are emerging as powerful model animals for generating novel insights into the mechanisms of NAFLD. Indeed, the analyses of NAFLD pathogenesis in these mutant fish models have identified important biological players, including Trappc11 and Cdipt, that have yet to be examined in mammals. NAFLD fish models and transgenic reporter fish have also been useful for drug testing, with n-3 PUFA, Tel, ezetimibe, taurine, certain vegetables, and translocator protein ligands all proven to be effective in reducing NASH pathology. In fact, some of these drugs, such as n-3 PUFA, have recently been recommended as potential NAFLD and NASH pharmacotherapies under the AASLD guidelines (Chalasani et al., 2012). We anticipate that NAFLD and NASH fish models will soon be utilized for additional therapeutic screening efforts.

The findings summarized in this Review highlight that natural and engineered fish models of NAFLD are useful tools for dissecting the still unknown mechanisms underlying non-alcoholic liver disease in humans. In addition, several new genomics technologies, such as ‘targeting induced local lesions in genomes’ (TILLING), zinc finger nuclease (ZFN) and transcription activator-like effector nuclease (TALEN), have been developed to perform more rapid targeted gene disruption in zebrafish and medaka (Huang et al., 2012). Together with transgenesis, the Cre-*loxP* system and various other inducible systems available for small fish, these genomic engineering tools are likely to accelerate the development of fish models of NAFLD that can be exploited to yield additional novel insights into this disease.
