# Correction: The expanding role of fish models in understanding non-alcoholic fatty liver disease

**DOI:** 10.1242/dmm.016022

**Published:** 2014-03

There was an error published in *Dis. Model. Mech*. **6**, 905–914 (2013). There is inappropriate paraphrasing/close copying of five unattributed review papers.

The authors agree that they were greatly influenced by the clarity and insight of the excellent reviews listed below and sincerely apologise for omitting to cite them, which was an unintentional mistake.

The references are listed below together with a description of the sections in which they should have been cited:

**Browning, J. D. and Horton, J. D.** (2004). Molecular mediators of hepatic steatosis and liver injury. *J. Clin. Invest*. **114**, 147–152. Citation missing from section on NASH pathogenesis: a ‘two-hit’ model.

**Dowman, J. K., Tomlinson, J. W. and Newsome, P. N.** (2010). Pathogenesis of non-alcoholic fatty liver disease. *Q. J. Med*. **103**, 71–83. Citation missing from section on NASH pathogenesis: a ‘two-hit’ model.

**Nivala, A. M.** (2011). Novel therapies for NAFLD. PhD thesis, Colorado State University. Citation missing from section on NASH pathogenesis: a ‘two-hit’ model.

**Schlegel, A.** (2012). Studying non-alcoholic fatty liver disease with zebrafish: a confluence of optics, genetics, and physiology. *Cell. Mol. Life Sci*. **69**, 3953–3961. Citation missing from Introduction and section on Mutant fish models of NAFLD and NASH.

**Stern, H. M. and Zon, L. I.** (2003). Cancer genetics and drug discovery in the zebrafish. *Nat. Rev. Cancer* **3**, 533–539. Citation missing from Figure 2 legend.

In accordance with COPE (Committee on Publication Ethics) guidelines, *Disease Models & Mechanisms* (DMM) recognises the difficulties that non-native writers face in correctly describing other authors’ work, but review articles are expected to provide an original synthesis of and commentary on previously published work: the words should be the authors’ own and all publications from whom they borrow ideas and words should be referenced.

DMM has carried out a thorough investigation into this matter and believe that the majority of the paper is original and appropriately referenced.

DMM is a member of COPE and is committed to maintaining the integrity of the published record and to publishing the most objective and unbiased scientific information possible. As such, DMM takes any claims of plagiarism very seriously and has a duty to its authors, editors and readers to investigate any such concerns. We list our publishing ethics policies clearly on our website and our policy on Originality and Plagiarism can be found at http://dmm.biologists.org/site/policies/publishing_ethics.xhtml#origin.

